# Development and evaluation of an ICT-based educational tool in home care nursing: A pilot qualitative study

**DOI:** 10.20407/fmj.2025-010

**Published:** 2025-11-05

**Authors:** Keisuke Iwase, Hironori Tsuzuki, Maki Ito, Tomomi Kawakami

**Affiliations:** 1 Faculty of Nursing, Fujita Health University, School of Health Sciences, Toyoake, Aichi, Japan; 2 School of Health Science, Department of Nursing, Toyohashi Sozo University, Toyohashi, Aichi, Japan

**Keywords:** Learning support application, Co-occurrence network, Assessment, Environment

## Abstract

**Background:**

In home care nursing, nurses are required to assess not only the care recipient’s condition but also their living environment. However, conventional paper-based case studies make it difficult for students to grasp the actual living environment.

**Objective:**

This study aimed to develop a learning support application (app) that combines 360-degree camera images with a paper-based case scenario and evaluate its usability among nursing students.

**Methods:**

Four areas inside the home of a single home care recipient were comprehensively photographed using a 360-degree camera. Thereafter, an app was developed to enable the users view the 360-degree images online by scanning with a smart device a QR code attached to a paper-based case scenario. To evaluate its usability, nursing students used the app and participated in semi-structured interviews. The data were analyzed using text mining techniques.

**Result:**

Text mining analysis revealed a co-occurrence network comprising four subgraphs. The representative keywords for each subgraph are as follows:

Subgraph 1: Consider, Home, Fall, Risk, Environment

Subgraph 2: Think, Danger, Imagination, Watch

Subgraph 3: Assessment, Learning support app, Residential environment, Home settings, Use

Subgraph 4: Life, See, Person, Situation

**Conclusion:**

The app supported students in understanding the home care environment and contributed to the development of care planning competence. Future studies should increase the number of cases and perform quantitative comparisons.

## Introduction

Japan is experiencing a rapidly aging population, and therefore, the importance of supporting home-based care has significantly increased.^1,2^ Advances in medical technology have diversified care settings, including hospitals, private homes, and long-term care facilities. Consequently, nurses are expected to view care recipients as individuals living within their everyday environments.^3^ However, as current nursing education remains largely hospital-centered, students may form a limited image of care recipients based primarily on clinical settings.

Home care nursing practicums offer students valuable opportunities to directly observe actual care environments. However, owing to geographical and time constraints, not all students are able to obtain such experiences. Although the COVID-19 pandemic has practically subsided, there persists a significant demand for flexible learning through remote education. Therefore, it is imperative to develop educational materials that enable students to experience the care environment from the perspective of individuals receiving care, outside of traditional clinical training settings.

Understanding the living environment of home care recipients is essential for developing appropriate nursing care plans, including fall risk assessment and formulation of strategies to improve activities of daily living (ADLs) based on behavioral patterns in daily life. Conventional audiovisual materials are effective in facilitating image formation for exercises and understanding real-life settings.^4,5^ However, flat photographs and standard 120-degree camera images offer limited fields of view, and videos restrict users’ control over their line of sight, making it difficult to grasp the overall environment. In contrast, 360-degree camera images allow users to shift their perspective and observe in any direction, enabling the comprehension of various aspects of the home care environment.

A systematic review by Pallavicini et al.^6^ concluded that learning using virtual reality (VR) is effective across various fields, including nursing. Similarly, a review study by Baysan et al.^7^ reported that the use of 360-degree videos contributes to nursing education as an alternative learning tool and enhances problem-solving skills. However, studies on the use of information and communication technology (ICT) in home care nursing education^8–10^ have mainly focused on instruction and live streaming. Examples of educational materials that integrate 360-degree images—reflecting the daily routines and movement patterns of home care recipients—with paper-based case scenarios remain scarce.

This practical report discusses the development of a learning support application (app) that integrates 360-degree images and a paper-based case scenario via cloud connectivity, with the aim of enhancing nursing students’ understanding of home care environments. Additionally, it presents the results of a qualitative user evaluation.

## Methods

The application was developed as a learning tool intended for application in community and home care nursing exercises for second- and third-year undergraduate nursing students. The intended learning objectives for students after using the app are as follows:

• To identify key risk factors in the living environment of home care recipients (e.g., steps in the bathroom, lack of handrails, inadequate lighting).
• To assess behavioral patterns in daily life and explain the key points of a nursing care plan based on the analysis (e.g., use of assistive devices, environmental modifications).

### App Development

#### Case Scenario Design

In collaboration with co-researchers, a paper-based case scenario was developed involving age-related conditions—cataracts and glaucoma. The scenario was designed to help students understand the importance of environmental modifications in the home care setting (Supplementary Material 1).

#### 360-Degree Image Capture

**Figure 1:**
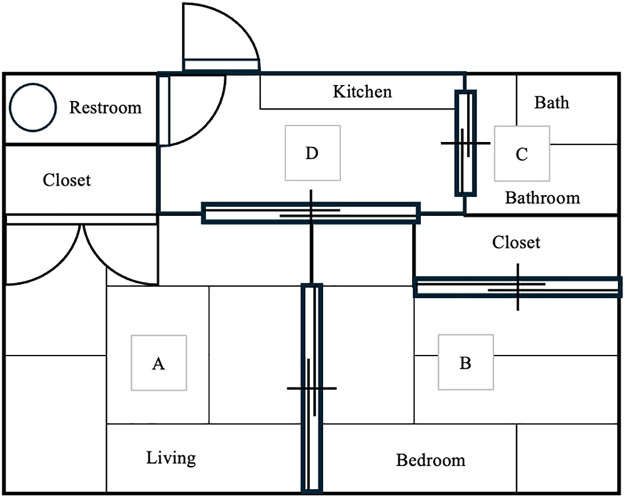
Floor plan Note: Locations A, B, C, and D indicate areas captured using a 360-degree camera.

**Figure 2:**
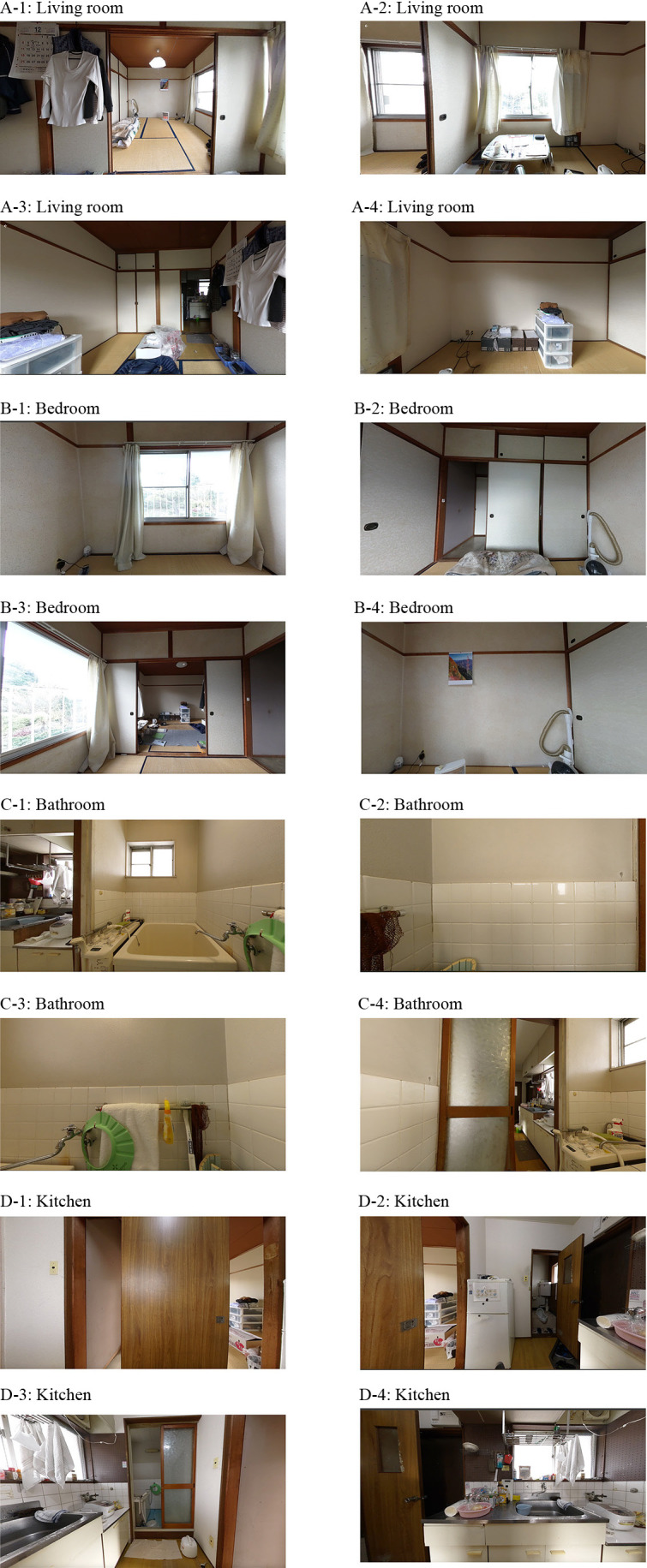
360-degree camera image

Using purposeful sampling, four areas: the living room, bedroom, bathroom, and entrance/restroom (Figures 1 and 2) inside the home of a care recipient who provided informed consent were comprehensively photographed using the THETA X (RICOH) 360-degree camera.

### Cloud Integration

A QR code was attached to the paper-based case scenario, enabling users to access the 360-degree images via a tablet or other smart device.

### User Evaluation

#### Participants and Study Period

The user evaluation was conducted between January and February 2023. Participants included four third- and fourth-year nursing students who had completed a home care nursing practicum in either the 2021 or 2022 academic year (one in 2021 and three in 2022). Participants were selected based on purposeful sampling and provided informed consent prior to participation. The timing of app use varied among the students, ranging from within one week to approximately one year after completing the practicum, depending on individual availability.

#### Learning Procedure

In this learning session, each student used the application individually in a private room, following the steps outlined below. The duration of the session was not measured, as the focus was on understanding the qualitative aspects of the learning process.

• *Orientation Session*

The researcher explained the purpose of the session and the operating procedures, including how to scan the QR code and perform basic screen navigation.

• *Case Scenario Review*

Students read the case scenario provided in Supplementary Material 1 to understand the background information of the case recipient.

• *Image Observation*

Students scanned the QR code using a tablet to access 360-degree images of the four areas of the home and observed them by freely navigating the view.

• *Nursing Process Development*

Students developed the nursing process by identifying nursing problems and formulating nursing care plans.

• *Semi-Structured Interview*

Following the session, a semi-structured interview was conducted using an interview guide (Supplementary Material 2) to explore students’ insights and the reasoning behind their judgments.

### Data Collection

To gain an in-depth understanding of the learning process and students’ insights during app use, this study employed semi-structured interviews as the primary method of qualitative evaluation.

### Data Analysis

**Figure 3:**
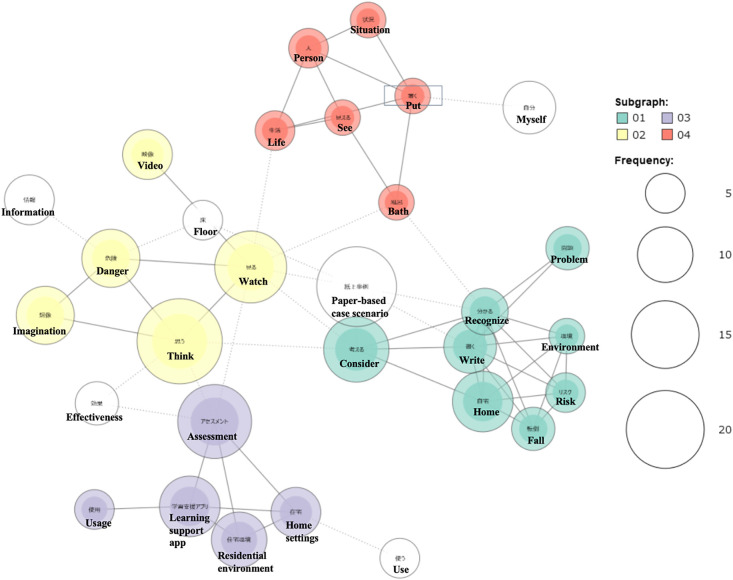
Co-occurrence network Note: The original text data were analyzed in Japanese. English translations of the extracted words are provided in this table.

Verbatim transcripts were analyzed using text mining with the free software KH Coder, based on a previous research.^11^ Relevant content was extracted from the transcripts in accordance with the study objectives. After preprocessing, word frequency counts and a co-occurrence network were generated (Figure 3). Reliability and validity were ensured through discussions among co-researchers. The analysis results are presented with English translations of the original Japanese data.

This evaluation was conducted as a pilot study with four participants to explore their experiences using the application. In future research, we intend to implement more rigorous evaluation methods, including the use of quantitative questionnaires and the inclusion of comparison groups.

## Ethical Considerations

This study was approved by the Research Ethics Committee of Fujita Health University (Approval No. HM22-268). Prior to participation, the purpose, content, data collection methods, and protection of personal information were explained both verbally and in writing to the care recipient and student participants. Written informed consent was obtained. Furthermore, the students were informed that there would be no disadvantages if they chose to not participate.

## Results

### App Development Outcome

With informed consent obtained from one care recipient for 360-degree image capture, the case scenario was completed. After verifying the QR code linkage and access functionality, the app was finalized.

### User Evaluation

The average duration of the semi-structured interviews conducted with four student participants was approximately 10 minutes each. Keywords related to user evaluation of the application are presented in angle brackets (< >) below.

### Extraction of Keywords Related to User Evaluation

The dataset used for analysis consisted of 40 sentences and 2,072 words. As presented in Table 1, frequently appearing words included <Think>, <Paper-based case scenario>, <Assessment>, <Watch>, and <Consider>.

### Co-occurrence Network

The co-occurrence network (Figure 3) revealed four subgraphs:

Subgraph 1: Co-occurring words such as <Consider>, <Home>, <Fall>, <Risk>, and <Environment> suggest that understanding the living environment may contribute to the assessment of fall risk.

Subgraph 2: Co-occurrence of <Think>, <Danger>, <Imagination>, and <Watch> indicates that the application may support the recall or recognition of potential hazards.

Subgraph 3: Words such as <Assessment>, <Learning support app>, <Residential environment>, <Home settings>, and <Use> were found to co-occur, suggesting that the app may facilitate students’ understanding of the home care environment.

Subgraph 4: The co-occurrence of <Life>, <See>, <Person>, and <Situation> suggests that the combined use of 360-degree images and a paper-based case scenario was perceived as beneficial.

## Discussion

### App Development

A novel aspect of this study lies in the development of an app that integrates a paper-based case scenario with 360-degree images via cloud connectivity, enabling students to observe home care environments from any perspective, regardless of time or location. According to a previous study,^12^ 360-degree images have the advantage of providing a panoramic view of the surroundings. This feature may expand learning opportunities by enabling students to actively identify subtle risk factors—for example, steps in the entrance or bathroom, presence or absence of handrails, and objects placed on the floor—that may be ignored in conventional still images or videos. However, concerns remain regarding disparities in students’ device and network environments, as well as data protection issues associated with cloud storage. Therefore, it is essential to provide thorough orientation on app operation, conduct prior verification of access conditions, and establish access control policies including data encryption.

### User Evaluation of the Application

Previous studies^13^ have demonstrated the effectiveness of ICT-based educational tools. In the present study, as shown in Subgraph 2 of Figure 3 (<Think>, <Danger>, <Imagination>, <Watch>), students tended to identify detailed points of attention and care, suggesting that 360-degree images are effective in improving their understanding of the care environment. Additionally, the co-occurrence of terms such as <Fall–Home–Environment> in Subgraph 1 of Figure 3 and the frequent appearance of <Home> and <Environment> in Table 1 indicate a tendency among students to concretely visualize the living environment of home care recipients. This tool may also contribute to the development of, what prior research^14^ refers to as, the “ability to observe, feel, and understand.” However, as no comparison group was included, future studies should strengthen the evaluation by conducting comparative research involving quantitative assessments.

As a future challenge, it will be necessary to expand the variety of case scenarios to enable students to learn about a broader range of living environments. This may enable them to appropriately assess the impact of the environment on care recipients and apply this understanding in practice.

### Limitation

This pilot study involved a small number of care recipients who participated in image capture and student participants, which limits the generalizability of the findings. Additionally, the timing of app use varied considerably—from one week to approximately one year after completion of the practicum—resulting in potential inconsistencies in the students’ learning experiences. Future studies should standardize the timing of app use and incorporate rigorous evaluations, including quantitative assessments. Furthermore, as semi-structured interviews were conducted immediately after app use, the influence of response bias (e.g., post hoc rationalization by participants) cannot be ruled out. Furthermore, the use of 360-degree images was limited to a single home environment, which rendered it insufficient to verify applicability across diverse living situations. Therefore, future research should expand the number of participants and case scenarios, compare the app with traditional educational materials, and introduce quantitative evaluation methods to comprehensively verify and refine the app’s effectiveness.

## Conclusion

This study developed an app that integrates 360-degree camera images with a paper-based case scenario. A user evaluation was conducted with four nursing students who had completed a home care nursing practicum. Text mining analysis revealed four co-occurrence subgraphs:

• Fall Risk Assessment (<Consider>, <Home>, <Fall>, <Risk>, <Environment>)
• Support for Hazard Awareness (<Think>, <Danger>, <Imagination>, <Watch>)
• Enhancement of Environmental Understanding (<Assessment>, <Learning support app>, <Residential environment>, <Home settings>, <Use>)
• Usefulness of Combining 360-Degree Images with Paper-based case scenario (<Life>, <See>, <Person>, <Situation>)

These findings suggest that the application contributes to improving students’ ability to assess and understand the home care environment. Future studies should expand the range of case scenarios and include quantitative evaluation to further verify its effectiveness.

## Figures and Tables

**Table 1:** Top 30 Most Frequent Words

Word	Frequency	Word	Frequency
Think	24	Information	8
Paper-based case scenario	21	Image	7
Assessment	18	Recognize	7
Watch	17	Effectiveness	6
Consider	14	Fall	6
Learning support app	12	Problem	6
Home	12	Risk	5
Danger	11	Use	5
Imagination	11	Usage	5
Residential environment	10	Floor	5
Myself	9	person	5
Write	9	Life	5
Video	8	Understanding	5
Home setting	8	Toilet	4
Actually	8	Environment	4

Note: The original text data were analyzed in Japanese. English translations of the extracted words are provided in this table.

## Supplementary Material

Supplementary Materials

PDF-Japanese
