# Supplementary material for: Development and evaluation of an ICT-based educational tool in home care nursing: A pilot qualitative study
Source: Fujita Med J. 2025 Nov 5;12(1):73–8. doi: 10.20407/fmj.2025-010 (PMC12865279; doi:10.20407/fmj.2025-010)
Supplement: Supplementary file 1 — Supplementary Materials [file fmj-12-073-s001.pdf]

Supplementary Material 1

A. Characteristics of the Individual Who Provided Consent for 360-Degree Image Capture

Attributes	Content
Age group	Early 70s
Sex	Male
Relevant medical conditions	Glaucoma, cataracts
Family members	Alone (in an apartment)
Other	Uses a white cane in daily life

Note: The 360-degree images were taken directly from the actual living environment of the individual described above.

B. Setting of the Paper-Based Case Scenario (Background information of the case character, edited and adapted for educational purposes)

Attributes	Content
Age group	72
Sex	Male
Relevant medical conditions	Postoperative status after laryngeal cancer surgery; cataracts and glaucoma (almost complete vision loss in the left eye). Tends to locate nearby objects by touch.
Family members	Alone (siblings live far away)
Activities of Daily Living	Uses a white cane; independent in shopping except on days when home-visit care is provided
Communication	Unable to speak owing to permanent tracheostomy; communicates through writing
Hobbies	Listens to the radio while drinking beer
Long-Term Care Insurance Certification Level	Level 1
Service Usage Status	Home-visit nursing (tracheostomy care and hygiene assistance)<br>Home-visit care (housework, medical appointments, etc.)
Independence Level of ADLs in Elderly People with Disabilities	A-2 – Partial assistance needed for tasks such as housekeeping and hygiene care
Cognitive Function	No decline

Case Overview

Mr. A lives alone on the second floor of an apartment. There is a 10–15 cm step at the entrance. The hallway is dimly lit, and the bathroom lacks handrails, making the tiled floor slippery. He uses a white cane to walk when he goes shopping, but often becomes unsteady because of steps and poorly lit areas.

Supplemental Material 2: Interview Guide

① Could you describe or visualize the living environment of a home care recipient in the context of home-visit nursing?

② Did the app help you form an image of the home care environment? Why or why not?

③ Were you able to use the app to assess the home care environment? Why or why not?

④ Do you think the app is effective for assessing home environments in a residential setting? Why or why not?

⑤ What improvements or enhancements would you suggest for the app?
