# Supplementary material for: Development and evaluation of an ICT-based educational tool in home care nursing: A pilot qualitative study
Source: Fujita Med J. 2025 Nov 5;12(1):73–8. doi: 10.20407/fmj.2025-010 (PMC12865279; doi:10.20407/fmj.2025-010)
Supplement: Supplementary file 2 — PDF-Japanese [file fmj-12-073-s002.pdf]

【背景】
在宅看護では、療養者の生活環境も含めた分析を求められるが、一般的な紙上事例では環境の把握が困難である。
【目的】
360°カメラ画像と紙上事例を組み合わせた学習支援アプリケーション（アプリ）を開発し、そのユーザー評価を実施した。
【方法】
在宅療養者の居室 4 カ所を 360°カメラで網羅的に撮影し、紙上事例に添付した QR コードをスマートデバイスで読み取ることで 360°画像を Web 上で閲覧できるアプリを作成した。アプリのユーザー評価を検証するために、学生に対し、アプリを利用した。その後、半構造化面接を行い、テキストマイニング分析を行った。
【結果】
テキストマイニング分析の結果、 4 つのサブグラフからなる共起ネットワークが構成された。それぞれのサブグラフと代表的な単語は、サブグラフ 1＜Consider＞、＜Home＞、＜Fall＞、＜Risk＞、＜Environment＞、サブグラフ 2＜Think＞、＜Danger＞、＜Imagination＞、＜Watch＞、サブグラフ 3＜Assessment＞、＜Learning support app＞、＜Residential environment＞、サブグラフ 4＜Life＞、＜See＞、＜Person＞で構成された。
【結論】アプリは学生の在宅環境理解を支援し、看護計画立案能力向上に資すると考えられる。今後は症例数拡大と定量比較を予定する。

キーワード：学習支援アプリケーション、共起ネットワーク、アセスメント、環境

はじめに

日本は高齢化が進展し、在宅療養支援の重要性が高まっている[1,2]。医療技術の進歩により、療養者の生活の場は自宅、介護施設など多様化しており、看護職は対象者を生活者として捉えることが求められている[3]。しかし、現状の看護教育は病院実習が大半を占め、患者像が先行しているという問題がある。

在宅看護学実習では、学生が実際の療養環境を直接観察する貴重な機会が得られるが、地理的および時間的な制約から、全員が十分に体験できるわけではない。さらに、Covid-19 禍は一旦落ち着きを見せつつあるものの、遠隔学習による柔軟な学びのニーズは依然高いため、臨地実習以外で生活者としての環境を体感させる教材の開発が急務である。

在宅療養者の生活環境を把握することは、転倒リスク評価や日常生活における行動パターンの理解を通じた ADL 改善計画の立案など、適切な看護計画を策定するために不可欠である。従来の視聴覚教材は演習のイメージ形成や現場理解に有効であると報告されている[4,5]が、平面写真や一般的な 120 度カメラでは視野が限定的であり、動画は視線操作が制限されるため環境の全体の把握には不十分である。一方、360°カメラ画像は視点移動が可能でユーザが任意の方向を観察できるため、在宅環境の多様な側面を把握できる利点がある。

Pallavicini らのシステマティックレビュー[6]では、バーチャルリアリティ（VR）を用いた学習が看護など多様な分野で有効であるとまとめている。また、Baysan らのレビュー研究[7]では、360°ビデオの使用が看護教育の代替や問題解決スキルの向上に寄与すると報告している。しかし、在宅看護学に ICT を活用した先行研究[8.9.10]は、指導や LIVE 配信が中心であり、在宅療養者の生活動線や日常生活を反映した 360°画像と紙上事例を連動させた教材の開発事例はほとんどない。

本実践報告では、在宅療養者の生活環境理解を促進することを目的に、360°カメラ映像と紙上事例をクラウド連携した学習支援アプリケーション（アプリ）を開発し、そのユーザー使用評価について報告する。

方法

本教材は、看護学部 2〜3 年次の地域・在宅看護学演習の学習ツールとしての導入を想定して開発した。アプリ使用後の学生の学習目標は以下の通りである。

・在宅療養者の生活環境から主要なリスク要因を特定できる（例：浴室の段差、手すりの不足、照明の暗さなど）

・日常生活における行動パターンを評価し、その分析結果を基に看護計画の要点を説明できる（福祉用具の適応、環境調整など）

アプリの開発

紙上事例設定

共同研究者間で高齢者特有疾患（白内障及び緑内障）の事例を設定し、在宅環境下での生活調整の重要性を学ぶ事例とした（Supplemental material 1）。

360°画像撮影

有意抽出法で同意を得た在宅療養者 1 名の居室 4 カ所（居間、寝室、浴室、玄関・トイレ）（図

1,図 2）を THETA X(RICOH)で網羅的に撮影した。

（図 1.2 挿入）

クラウド連携

紙上事例に QR コードを添付し、タブレットなどで 360° 画像を参照可能とした。

ユーザー使用評価

研究対象・調査期間

本評価は 2023 年 1〜2 月に実施し、対象は 2021 年度または 2022 年度に在宅看護学実習を修了した 3〜4 年次学生 4 名（うち 2021 年度修了 1 名、2022 年度修了 3 名）である。対象者は有意抽出法により選定し、同意を得た上で実施した。対象学生 4 名のアプリ視聴タイミングは、個々の都合により在宅看護学実習修了後 1 週間以内から約 1 年後まで幅があった。

学習手順

本学習では、学生は個室で個別に以下の手順でアプリを使用し、学習を進めた。所要時間は測定せず、質的な学習プロセスの理解に重点を置いた。

・事前オリエンテーション

研究者から本学習の目的と操作手順（QR コードの読み取り方法、画面操作の基本）について説明を受ける

・紙上事例の熟読

Supplementary Material 1 に示した事例を読み、事例人物の背景情報を把握する。

・360° 画像の閲覧

タブレットで、事例の QR コードを読み取り、居室 4 ヵ所の 360° 画像を自在に操作しながら観察する。

・観察結果の記録

看護過程を展開し、看護問題や看護計画を立案する。

・半構造化面接

インタビューガイド（supplemental material 2）に沿って、学習における気づきや判断根拠を深掘する。

データ収集方法

本研究では、アプリ使用時の学習プロセスや気づきの質的側面を深く理解することを目的に、半構造化面接によるインタビューを評価手法として採用した。

データ分析方法

逐語録は無償の KH Coder を用いて先行研究[11]に基づくテキストマイニング分析を実施した。目的に沿った内容を逐語録から抽出し、前処理後に単語の出現回数の集計及び共起ネットワーク（図 3）の作成を行い、共同研究者間で信頼性と妥当性を確認した。分析結果は日本語データに英語翻訳を付して報告した。本評価はパイロットスタディとして、4 名によるユーザー使用評価で

ある。今後は、定量的質問紙調査や比較群の設定を含む厳密な評価手法の導入を計画している。

倫理的配慮

本研究は、藤田医科大学医学研究倫理委員会の審査にて学長の承認（HM22-268）を得た上で実施した。撮影対象者及び学生には、口頭と文書で研究目的、内容、個人情報保護、データ収集方法等を説明し、書面同意を得た。なお、学生には参加拒否による不利益が生じないことも説明した。

結果

アプリの開発成果

対象者 1 名から撮影同意を得て事例作成を完了。QR コード連携・リンク検証を経てアプリを完成させた。

ユーザー使用評価

対象学生 4 名の平均面接時間は約 10 分であった。以下、抽出されたアプリの評価に関する語句を＜　＞で示す。

アプリのユーザー使用評価に関する語句の抽出

分析対象データは、40 文、2,072 語であった。頻出語句（表１）では、＜Think＞、＜Paper based patient＞、＜Assessment＞、＜Watch＞、＜Consider＞などが上位を占めた。

（表 1 挿入）

共起ネットワーク

共起ネットワーク（図 3）では 4 つのサブグラフが示された。

サブグラフ 1：＜Consider＞、＜Home＞、＜Fall＞、＜Risk＞、＜Environment＞などの語句が共起していることが確認され、生活環境の把握が転倒リスクの評価に役立つ可能性が示唆された。

サブグラフ 2：＜Think＞、＜Danger＞、＜Imagination＞、＜Watch＞などの語句が共起していることが確認され、アプリの使用が危険性の想起を支援することが示唆された。

サブグラフ 3：＜Assessment＞、＜Learning support app＞、＜Residential environment＞、＜Home settings＞、＜Use＞などの語句が共起していることが確認され、アプリが生活環境の理解促進に繋がることが示唆された。

サブグラフ 4：＜Life＞、＜See＞、＜Person＞、＜Situation＞などの語句が共起していることが確認され、360°画像と紙上事例併用が有用であることが示唆された。

（図 3 挿入）

考察

アプリの開発

本研究の新規性は、紙上事例と 360° 画像をクラウド連携し、学生が場所や時間を問わず自由視点で在宅環境を観察できるアプリを構築した点にある。先行研究 12 によれば、360° 画像は全周を俯瞰できるため、従来の静止画や動画では見落としがちな玄関や浴室などの段差、浴室の手すり、床に置いてある物品などの微細なリスク要因を主体的に発見する学習機会を大きく拡張する可能性がある。一方、学生の端末環境やネットワーク状況の格差、クラウド保存に伴う情報保護の課題もあるため、操作オリエンテーションの充実、アクセス環境の事前確認、データ暗号化を含むアクセス制御ポリシーの策定などが不可欠である。

アプリのユーザー使用評価

先行研究[13]は、ICT 教材の有効性を示している。本研究では、図 3 のサブグラフ 2（＜Think＞、＜Danger＞、＜Imagination＞、＜Watch＞）に示されたように、細やかな配慮すべき点や注意点を抽出する傾向が示され、360° 画像が環境理解を深める有効性を示唆した。また、図 3 のサブグラフ 1（＜Fall–Home–Environment＞連関）および表 1 の頻出語句（＜Home＞、＜Environment＞）から、学生が在宅療養者の生活環境を具体的にイメージする傾向が示された。加えて、先行研究[14]が指摘する「見て感じて把握する力」の育成にも寄与する可能性がある。ただし、対象群比較は行っていないため、今後は定量評価を含む比較研究を通じ、効果検証を強化する必要がある。

今後の課題として、療養者の多様な環境を学ぶために事例を拡充し、様々な生活環境を学習できる環境を整える必要がある。これにより、生活環境が療養者に与える影響を考慮した支援を適切に判断し、実践に繋げることが期待される。

研究の限界

本研究はパイロットスタディであり、撮影協力者および参加学生の人数が非常に少なく、知見の一般化に限界がある。また、アプリ実施時期は実習終了後 1 週間～約 1 年と幅があり、学習体験の一貫性を欠いた可能性がある。今後は視聴時期を統一し、定量的評価を含む厳密な検証を行う必要がある。加えて、アプリ使用直後に半構造化面接を行ったため、回答には Response Bias（回答者の事後的脚色）の影響も排除できない。さらに、単一住居の 360° 画像撮影に留まるため、多様な生活環境への適用性検証は不十分である。今後は、対象者数や撮影事例の拡大、従来教材との比較、および定量的評価手法の導入により、アプリの有効性をより包括的に検証・改善する必要がある。

結論

本研究では、360° カメラ画像と紙上事例を組み合わせたアプリを開発し、在宅看護学実習を修了した学生 4 名を対象にユーザー使用評価を行った。テキストマイニング分析から、以下の 4 つの共起サブグラフが抽出された。

・転倒リスクの評価（＜Consider＞、＜Home＞、＜Fall＞、＜Risk＞、＜Environment＞）
・危険性の想起支援（＜Think＞、＜Danger＞、＜Imagination＞、＜Watch＞）
・生活環境理解促進（＜Assessment＞、＜Learning support app＞、＜Residential environment＞、

＜Home settings＞、＜Use＞）

・360° 画像と紙上事例併用の有用性（＜Life＞、＜See＞、＜Person＞、＜Situation＞）

これらは、本アプリが在宅看護における環境把握およびアセスメント理解の向上に寄与し得ることを示唆している。今後は、対象症例の拡大と定量的検証を通じ、さらなる効果の検証が求められる。

利益相反

本研究は、藤田医科大学教員研究助成費により実施し、利益相反は認められなかった。

謝辞

本研究にご協力いただいた全ての方に感謝する。

引用文献

1．Ministry of Health, Labour and Welfare. Waga kuni no jinkou ni tsuite(About the Population of Our Country) (in Japanese).
<https://www.mhlw.go.jp/stf/newpage_21481.html >(Accessed March 4, 2025)

2．Ministry of Health, Labour and Welfare. Chiiki Houkatu Kea shisutemu(Community-Based Integrated Care System) (in Japanese).
<https://www.mhlw.go.jp/stf/seisakunitsuite/bunya/hukushi_kaigo/kaigo_koureisha/chiiki-houkatsu/index.html > (Accessed April 18, 2024)

3．Ministry of Health, Labour and Welfare. Kango Kiso Kyouiku Kentoukai Houkokusho(Report of the Nursing Fundamental Education Review Meeting);2019 (in Japanese).
< https://www.mhlw.go.jp/content/10805000/000557411.pdf > (Accessed April 18, 2024)

4．Matsui S, Masatoki K, Sugino H, Murata S, Nakai Y. Evaluating E-learning Materials for the Development of Nursing Skills in Adult Nursing. FPU Journal of Nursing Research 2015;12: 63-71 (in Japanese).

5．Yamaguchi Y, Murase M, Matsumoto K, Serikawa Y, Tanaka K. A report on educational methods in on-campus training using videos due to reduction in the on-site nursing practical training time: Analysis of students' responses to questionnaire in home care nursing practical training. Journal of Kumamoto Health Science University 2021;18: 103-15(in Japanese).

6．Pallavicini F, Pepe A, Clerici M, Mantovani F. Virtual Reality Applications in Medicine During the COVID-19 Pandemic: Systematic Review. JMIR Serious Games. 2022 Oct 25;10(4):e35000. doi: 10.2196/35000. PMID: 36282554; PMCID: PMC9605086.

7．Baysan A, Çonoğlu G, Özkütük N, Orgun F. Come and see through my eyes: A systematic review of 360-degree video technology in nursing education. Nurse Educ Today. 2023 Sep;128:105886. doi: 10.1016/j.nedt.2023.105886. Epub 2023 Jun 17. PMID: 37390520.

8．Kurihara R. The Outcomes of Active Learning Using the e-Learning System in Home Care

Nursing Practices. The journal of Faculty of Health and Welfare Science, Asahikawa University 2020; 12:35-40(in Japanese).

9. Okada M, Katayama Y, Suwa A. Challenging and evaluating home care nursing practices using an interactive online learning program in a nursing university-implementing the new education system in the context of COVID-19 pandemic-. Journal of Kagawa Prefectural University of Health Sciences 2021;12: 57-65(in Japanese).

10. Sakamoto T, Yamagata M. Basic Home Care Nursing Education Complementing Clinical Practice Using Information and Communication Technology(ICT): Practical Training Content and Its Organization under Restricted Conditions. Kawasaki Medical Welfare Journal 2022;32: 257-63(in Japanese).

11. Kitano Y, Hori M, Muto S. Tashokusyurenkei ni okeru gakusei no manabi: Text mining tool ni yoru kougi no manabi no bunseki kara(Student Learning in Interprofessional Collaboration: Analysis of Lecture Learning Using Text Mining Tools). Bulletin of Department of Nursing, Asahi University 2023;9: 19-23(in Japanese).

12. Tsuhako K, Kinoshi T, Sakanashi S, Inoue,H, Sone, E, Miyako J, Yanagi S, Nakagawa K, Saito S, Kanagawa Y, Katsurahara T, Kozakai K, Omori S, Tanaka H. How the ICT-based sports event medical support system should work. The Annual Reports of Health, Physical Education and Sport Science 2023;41: 111-21(in Japanese).

13. Webb L, Clough J, O'Reilly D, Wilmott D, Witham G. The utility and impact of information communication technology (ICT) for pre-registration nurse education: A narrative synthesis systematic review. Nurse Educ Today 2017; 48: 160-71.

14. Taguchi R, Kawahara C, Nishi R, Sueda C. Basic abilities required to perform home care nursing planning: an interview of expert home-visit nursing station managers. Kyoritsu Journal of Nursing 2015; 2: 1-9(in Japanese).
